# Two closely related β-1,2-xylosyltransferases differentially impact cryptococcal glycan synthesis

**DOI:** 10.1128/mbio.00806-26

**Published:** 2026-09-29

**Authors:** Daphne Boodwa-Ko, J. Stacey Klutts, Kazuhiro Aoki, Michael L. Skowyra, Indrani Bose, Daniel P. Agustinho, Mayumi Ishihara-Aoki, Michael Tiemeyer, Tamara L. Doering

**Affiliations:** 1 Department of Molecular Microbiology, Washington University School of Medicine 169015, St. Louis, Missouri, USA; 2 Complex Carbohydrate Research Center, Athens, Georgia, USA; Yonsei University, Seoul, Republic of Korea

**Keywords:** *Cryptococcus neoformans*, glycosylation, glycosyltransferase, *O*-glycan, xylose, xylosyltransferase

## Abstract

*Cryptococcus neoformans* is an opportunistic fungal pathogen that causes pulmonary infection in immunocompromised patients, which in severe cases leads to fatal meningoencephalitis. *Cryptococcus* exhibits unique glycobiology that plays important roles in pathogenesis. Unlike model yeast and other common fungal pathogens, *Cryptococcus* incorporates xylose, a five-carbon monosaccharide, into its glycans. One trimer motif, which consists of xylose in β-1,2 linkage to the reducing mannose of an α-1,3-mannose dimer, occurs in key cryptococcal glycoconjugates that include protein *N-* and *O*-linked glycans, glycosylinositol phosphorylceramides (GIPCs), and the capsule polysaccharides glucuronoxylomannan (GXM) and glucuronoxylomannogalactan (GXMGal). We previously identified cryptococcal β-1,2-xylosyltransferase 1 (Cxt1), which catalyzes the formation of this motif in GIPCs, GXM, and GXMGal. Here, we report the discovery of a second enzyme, cryptococcal β-1,2-xylosyltransferase 2 (Cxt2). Through characterization of cells that lack one or both corresponding genes (*CXT1* and *CXT2*), we have dissected the biological roles of these enzymes, which are overlapping but not identical. Notably, Cxt1 and Cxt2 colocalize in the Golgi, influence the capsule in a strain-dependent manner, and together are responsible for all xylose addition to *O*-glycans. Overall, our work highlights the unique roles of these two enzymes and fills a gap in our understanding of cryptococcal glycan synthesis.

**IMPORTANCE:**

*Cryptococcus neoformans* is an opportunistic fungal pathogen that causes almost 150,000 deaths each year worldwide. *Cryptococcus* synthesizes unique glycan structures that play important roles in its biology and pathogenesis. One abundant component of these structures is xylose, a five-carbon monosaccharide. Because xylose is not used by many fungal organisms, including model yeast, we have limited information about how cells add it to their glycans. Here we report a xylosyltransferase enzyme that performs this function, and we characterize specific biological roles of this protein and a closely related one we discovered earlier. We find that these proteins together perform all detectable xylose addition to an important class of protein-linked glycans (*O*-glycans). They also both participate in other synthetic processes, although this varies with the specific enzyme and strain background. These results contribute to our understanding of cryptococcal glycan synthesis and underscore the importance of testing multiple background strains.

## INTRODUCTION

*Cryptococcus neoformans* is an opportunistic fungal pathogen found ubiquitously in the environment. This haploid yeast primarily infects immunocompromised patients, such as HIV/AIDS patients and transplant recipients (1). *C. neoformans* first enters the lungs as spores or desiccated yeast and establishes a pulmonary infection. It can subsequently disseminate to the central nervous system, where it causes cryptococcal meningoencephalitis. There are nearly 200,000 cases of cryptococcal meningoencephalitis annually, leading to approximately 150,000 deaths (1). Current treatment options for this devastating disease are limited (2), although new therapies are being explored.

*C. neoformans* is characterized by a polysaccharide capsule that surrounds its fungal cell wall. The capsule consists of two large polysaccharides: glucuronoxylomannan (GXM) and glucuronoxylomannogalactan (GXMGal) (3). GXM, which makes up ~90% of capsule mass, consists of an α-1,3-mannan backbone with variable glucuronic acid and xylose substitutions (Fig. S1A). GXMGal is composed of an α-1,6-galactan backbone bearing galactomannan side chains that are substituted with glucuronic acid and xylose (Fig. S1B) (4, 5). The capsule markedly expands when *Cryptococcus* enters a mammalian host or is grown *in vitro* under conditions that mimic the host environment (6–8). Capsule polysaccharides are essential for virulence and play important roles in modulating the host immune response (9–15).

*C. neoformans* has historically been categorized into four serotypes (A–D) (3, 4, 16–18). Serotypes B and C correspond to *Cryptococcus gattii* and *Cryptococcus bascillisporus*, respectively, while serotypes A and D correspond to two varieties of *C. neoformans*, var. *grubii* and var. *neoformans*. These have also been named *C. neoformans* and *Cryptococcus deneoformans* (19), the nomenclature we use below. *C. neoformans* is the most common *Cryptococcus* species in environmental, clinical, and veterinary isolates and makes up 63% of all collected isolates. *C. deneoformans* is much less common, accounting for 4%–5% of isolates (19). This disparity may reflect differences in cellular properties and virulence, with *C. neoformans* generally more thermotolerant and more virulent than *C. deneoformans* (19, 20). Their capsule GXM composition also differs: *C. neoformans* GXM typically exhibits more xylose substitutions than *C. deneoformans* GXM (4). Despite these distinctions, the two species are closely related, with near-complete synteny and 85%–90% nucleotide identity (21). Both have been used extensively in cryptococcal research.

Apart from the capsule, *C. neoformans* synthesizes multiple other glycoconjugates, including glycolipids, glycoproteins, and cell wall glycans. Generating these structures requires the synthesis and transport of nucleotide sugars, which act as donors for many glycan biosynthetic processes (22). The transfer of saccharide moieties from nucleotide sugar donors to specific acceptor molecules is catalyzed by glycosyltransferases (GTs). These critical enzymes are generally specific for the donor and acceptor molecules and form specific glycosidic linkages. Importantly, while amino acid sequences can be predicted to encode GTs, their specific enzymatic activity cannot be accurately determined without biochemical assays. Despite significant research on cryptococcal GTs (22, 23), we do not yet know the full array of enzymes that build cryptococcal glycoconjugates.

Xylose is a five-carbon monosaccharide that does not occur in the model yeast *Saccharomyces cerevisiae* (24, 25), although it has been reported in *C. neoformans* and a handful of other fungal species: the basidiomycetes *Trichosporon asahii* and *mucoides* and the ascomycete *Paracoccidioides brasiliensis* (26–29). It is notably abundant in cryptococcal capsule polysaccharides, *N-* and *O-*linked glycoproteins, and glycosylinositol phosphorylceramides (GIPCs). In many of these, xylose occurs in a trimer motif linked β-1,2 to α-1,3-linked mannose, including in capsule polysaccharides, *N-* and *O-*linked glycoproteins, and glycosphingolipids (Fig. S1, bold text) (26–30).

In *Cryptococcus*, xylose modifications play significant roles in virulence, immune modulation, and cell surface integrity (31–35). The absence of proteins that participate in the synthesis or transport of UDP-xylose, the specific donor for xylosyltransferase enzymes, leads to altered capsule structure and dramatically reduced virulence (31, 32). Conversely, mutants with increased proportions of xylose-containing *N-* and *O*-linked glycans have enhanced immunogenicity compared to their non-xylosylated counterparts (34, 35). Defining the enzymes that create these structures is thus important for understanding this pathogen.

In search of capsule synthetic enzymes, we previously identified a cryptococcal β-1,2-xylosyltransferase 1 (Cxt1). This adds xylose in β-1,2 linkage to the reducing mannose residue of α-1,3-mannobiose *in vitro* (36), forming the common triad noted above. Deletion of the corresponding gene (*CXT1*) eliminates GIPC xylosylation and yields reduced xylose in GXM and GXMGal (37, 38) but does not fully eliminate capsule xylosylation. This suggests the existence of at least one other xylosyltransferase enzyme with similar activity. Here, we report on such an enzyme, which we have named cryptococcal β-1,2-xylosyltransferase 2 (Cxt2), and use biochemical, phenotypic, and imaging approaches to define its role in cryptococcal biology. We also demonstrate the role of both enzymes in *O*-glycan synthesis.

## RESULTS

### *C. neoformans* expresses a second β-1,2-xylosyltransferase

**Fig 1:**
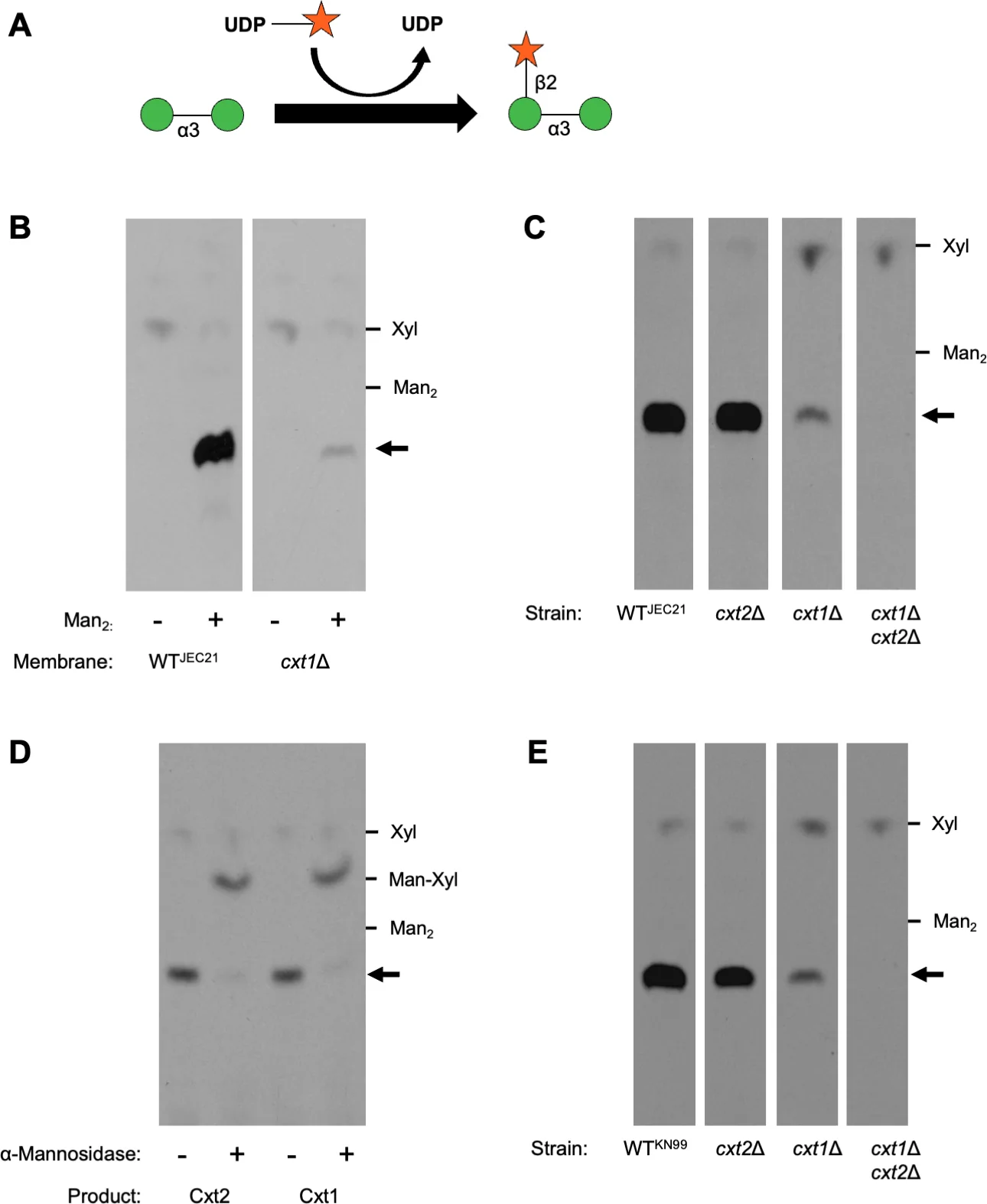
β-1,2-Xylosyltransferase activities. (**A**) Schematic of the β-1,2-xylosyltransferase activity assay. Cxt1 catalyzes the transfer of xylose from UDP-xylose to α-1,3-mannobiose (Man_2_), forming a β-1,2-linkage as shown. Green circle, mannose; orange star, xylose. (**B–E**) Autoradiographs of assay products resolved by TLC. Migration positions of non-radiolabeled standards are indicated at the right; the black arrows indicate the migration position of the Cxt1 product. WT, wild-type strain; the superscript indicates the strain background, either JEC21 (*C. deneoformans*) or KN99 (*C. neoformans*). (**B, C, and E**) Washed membranes from the indicated strains were assayed as described in Materials and Methods. (**D**) Reaction products from assays as in panel **C** were mock treated or incubated with Jack Bean α-mannosidase (see Materials and Methods) before TLC.

We previously identified Cxt1 as a cryptococcal β-1,2-xylosyltransferase responsible for adding xylose to capsule polysaccharides and GIPCs (36–38). *In vitro*, Cxt1 catalyzes the transfer of xylose from UDP-xylose to the reducing mannose of an α-1,3-mannobiose (Man_2_) acceptor, forming a β-1,2 linkage (Fig. 1A). This product is readily observed in assays with wild-type strains (WT) (Fig. 1B, left panel). Interestingly, we still observed a small amount of this product in assays of membranes from *cxt1*∆ deletion strains (Fig. 1B, right). We speculated that its synthesis was catalyzed by another *C. neoformans* enzyme, which we wished to identify.

One candidate for a potential second β-1,2-xylosyltransferase was *CAP5* (gene ID: CND04030)*,* the closest homolog of *CXT1* in the *C. neoformans* genome with 40% amino acid identity and 56% similarity (36, 39). Targeting this gene by RNAi in a *cxt1*∆ background eliminated the residual xylosyltransferase activity, while targeting another homolog, *CAP4* (gene ID: CNB02330), did not (Fig. S2). We hypothesized that the *CAP5* sequence encoded an additional β-1,2-xylosyltransferase.

To test this hypothesis, we deleted the *CAP5* coding sequence, generated otherwise isogenic wild-type and mutant strains by mating, and assessed β-1,2-xylosyltransferase activity. While deletion of this gene alone (strain labeled *cxt2*∆) had little or no effect on the amount of product formed (Fig. 1C; compare the first two lanes), its deletion in the *cxt1*∆ background eliminated the residual assay product previously observed in that mutant (Fig. 1C; compare the last two lanes). Based on these results, which were consistent with our RNAi experiments, we concluded that this sequence encoded a second β-1,2-xylosyltransferase and renamed it *CXT2*, for cryptococcal xylosyltransferase 2.

Our prior analysis of the Cxt1 reaction product identified it as α-1,3-mannobiose with β-1,2-xylose on the reducing mannose (36). Because the low abundance of the Cxt2 product (Fig. 1C) made it challenging to isolate sufficient material for such analysis, we instead compared the chromatographic behavior of the two assay products. Their comigration on a sensitive thin-layer chromatography (TLC) system, which resolves multimers of different sizes as well as of different linkages (40, 41), already suggested they had the same structure. To complement these studies, we isolated the Cxt1 and Cxt2 reaction products from *cxt2*∆ and *cxt1*∆ strains, respectively, and treated them with Jack Bean mannosidase, which cleaves α-mannose linkages, including α-1,3. Each of the resulting digestion products, which we know for Cxt1 is Xyl-β-1,2-Man (36), also comigrated (Fig. 1D). Together, these results show that both Cxt1 and Cxt2 are *C. neoformans* β-1,2-xylosyltransferases.

### Cxt1 and Cxt2 are well conserved between *C. neoformans* serotypes

Although we performed our early experiments in the *C. deneoformans* strain JEC21, we were interested in whether Cxt1 and Cxt2 were conserved in *C. neoformans*, which causes most human disease and is more virulent. Sequence alignments showed that Cxt1 from JEC21 and *C. neoformans* strain KN99 (42) are 93.23% identical and 96.11% similar; Cxt2 is 88.26% identical and 92.18% similar (Fig. S3A and B). We also assessed structural similarity by calculating the template modeling (TM) score based on the AlphaFold 3 predicted structures of each protein (Fig. S3C and D). The TM score for JEC21 and KN99 Cxt1 was 0.95, suggesting high structural similarity. The Cxt2 score was lower (0.80) but still well within the range for structurally similar proteins (0.5–1.00) (43).

When we assayed deletion mutants engineered in KN99, we observed the same product and pattern of β-1,2-xylosyltransferase activity as we had in the *C. deneoformans* strain (Fig. 1E). We conclude that the Cxt enzymes make the same product in both species, with Cxt1 producing the majority of the xylosylated product *in vitro*, while Cxt2 produces a smaller fraction.

### β-1,2-Xylosyltransferases impact temperature sensitivity in JEC21

**Fig 2:**
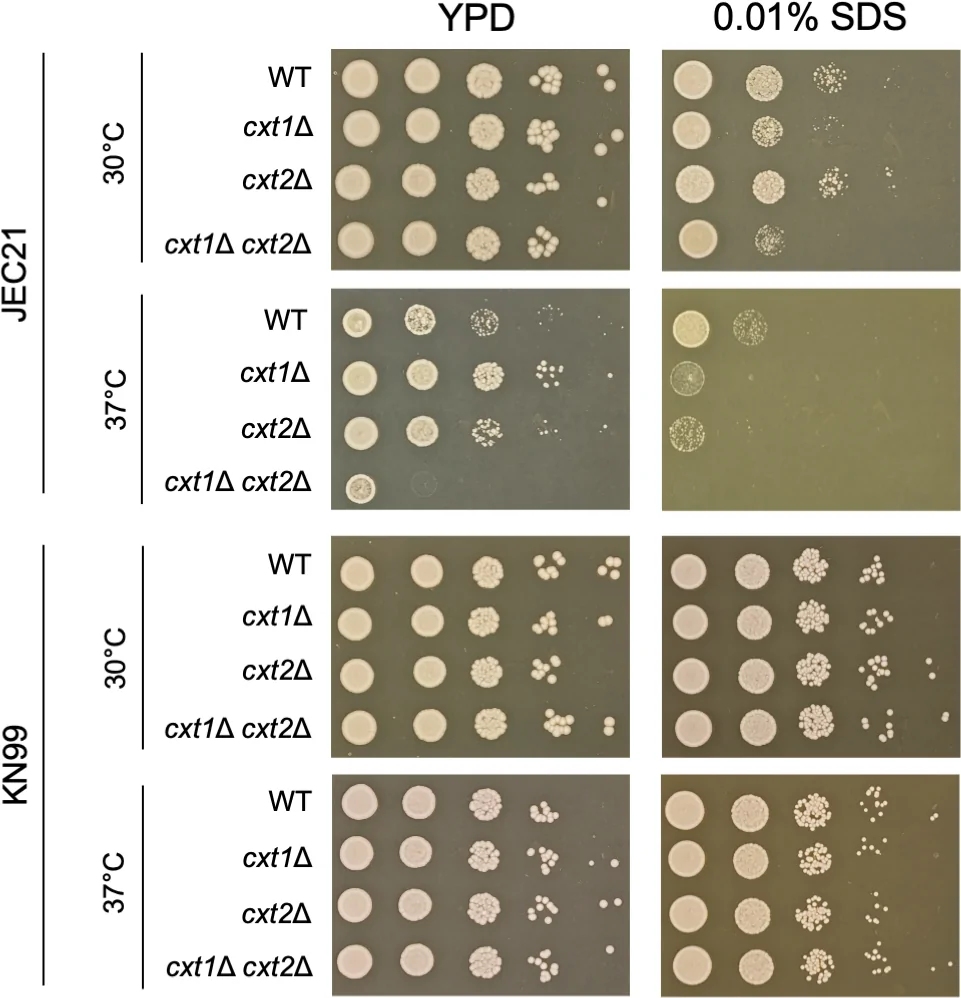
Impact of β-1,2-xylosyltransferases on growth *in vitro*. The indicated strains were plated in 10-fold serial dilutions on YPD agar medium ± 0.01% SDS and incubated for 72 h as shown. The experiment shown is representative of three similar studies.

To better understand the biological roles of Cxt1 and Cxt2, we compared the growth of single- and double-deletion mutants to wild-type cells in various stress conditions. The JEC21 *cxt1*∆ *cxt2*∆ strain showed increased sensitivity to 37°C and could not grow at this temperature in the presence of 0.01% SDS, a membrane stressor (Fig. 2). The temperature sensitivity was not exacerbated by high salt, which induces osmotic stress, or calcofluor white (CFW), a cell wall stressor (Fig. S4). The single mutants in this background showed a mixture of phenotypes under stress. Compared to wild type, *cxt1*∆ grew more robustly at 37°C but was more sensitive to SDS. The *cxt2*∆ mutant resembled wild type in most conditions, although it, like *cxt1*∆, grew worse than wild type in the combined stress of SDS and 37°C. It also grew slightly less well than wild type in DMSO and CFW at that temperature (Fig. S4). In contrast, all KN99 strains grew comparably under all these conditions. Since *C. neoformans* strains are more temperature resistant than *C. deneoformans*, we repeated these experiments at 39°C, but observed no difference in growth among KN99 strains (data not shown).

### Cxt1 but not Cxt2 impacts glycosphingolipid synthesis

The SDS sensitivity of the JEC21 mutants suggested a defect in cell membranes, consistent with our earlier finding that Cxt1 catalyzes the addition of xylose to GIPCs (37). To assess any effect of Cxt2 on this process, we isolated GIPCs from our JEC21 strain set, as well as from a *uxs1*∆ control strain that cannot form UDP-xylose and therefore lacks all glycosphingolipid xylosylation (30). High-performance TLC of these samples confirmed that the GIPC profile of *cxt1*Δ cells matches that of the *uxs1*∆ control, indicating they contain no xylose (Fig. S5; note loss of xylosylated-GIPC and accumulation of non-xylosylated-GIPC); results for the double mutant *cxt1*Δ *cxt2*Δ were similar. In contrast, *cxt2*Δ phenocopies wild type, suggesting that Cxt2 has no role in this process. These results are consistent with previous findings that Cxt1 is solely responsible for GIPC xylosylation. However, it also suggests some other reason for the sensitivity of *cxt2*∆ to the combination of SDS and 37°C.

### Cxt1 and Cxt2 add xylose to *O*-linked, but not *N*-linked, glycans

Some *Cryptococcus* mutants defective in *N-* and *O-*linked glycosylation are sensitive to SDS and temperature (34, 44–46), and studies of *N*- and *O*-glycans in *C. neoformans* suggest that both contain xylose (44, 45, 47). We therefore wondered whether some of the altered growth of our *cxt* mutants might be explained by defects in protein glycosylation.

**Fig 3:**
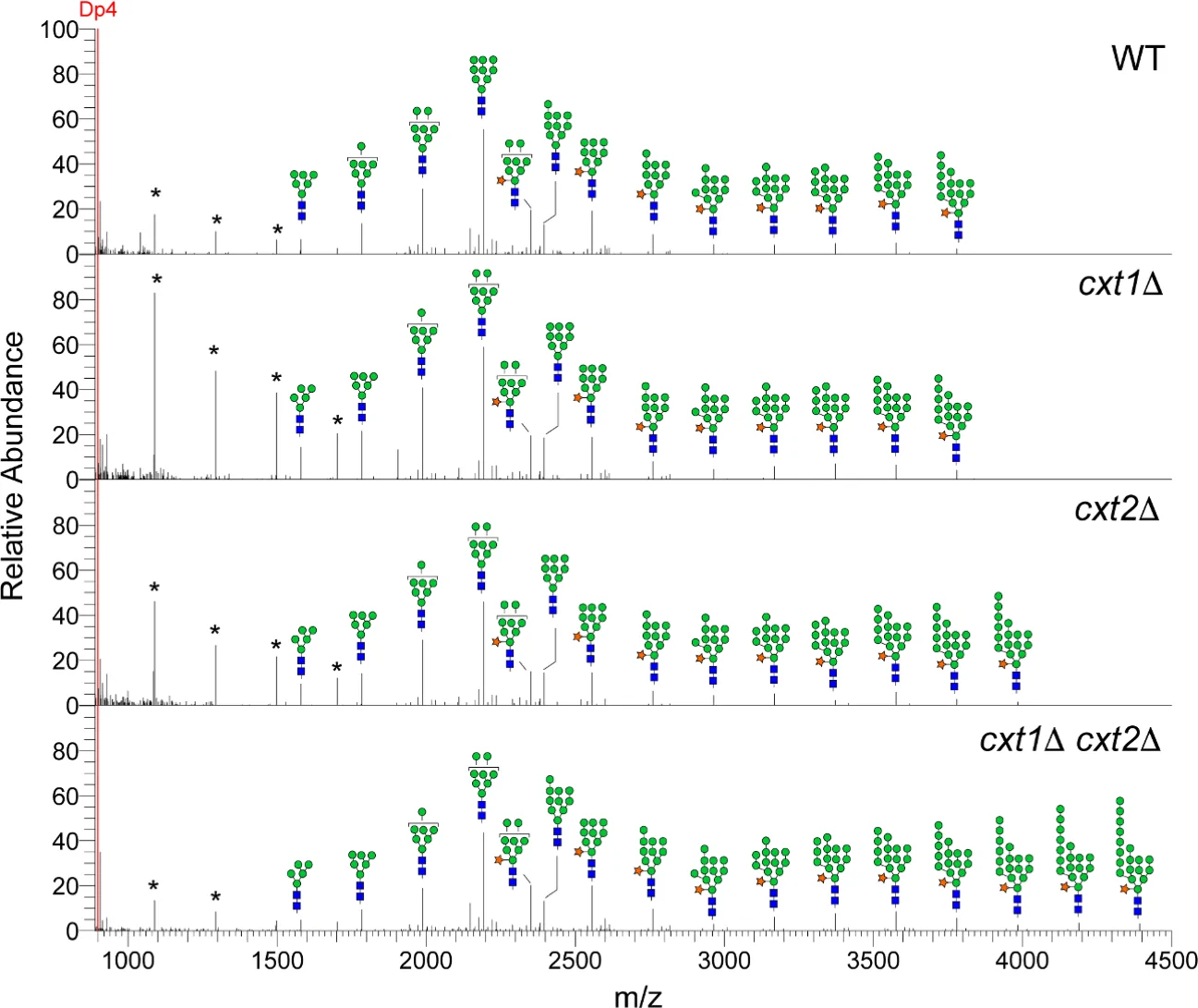
Mass spectrometry analysis of *N*-linked glycans from the indicated strains. DP4 (10 pmol), a maltotetraose standard, was spiked into the *N*-glycan samples for direct comparison. Glycan structures are shown above the corresponding peak; specific *m*/*z* values and quantitation are listed in Table S1. *, common hexose ladder contaminant; blue square, *N*-acetylglucosamine; green circle, mannose; orange star, xylose.

*N-*linked glycans consist of a branched mannose structure linked to asparagine via a chitobiose core of two *N*-acetylglucosamine residues. In *C. neoformans*, xylose occurs in β-1,2 linkage on the first mannose beyond the core (Fig. S1D). While Cxt1 has been reported to have no direct role in the xylosylation of *N-*glycans (44), Cxt2’s role in this process is not known. When we analyzed *N*-linked glycans in our KN99 strain set by mass spectrometry, the overall profiles were not significantly altered, including the overall abundance of xylose modification (Fig. 3). These results suggest an overall minor impact of Cxt1 and Cxt2 on *N*-glycan xylose modification, consistent with earlier observations on Cxt1 (44). However, compared to wild type or either single mutant, the *cxt1*Δ *cxt2*Δ mutant did exhibit a preponderance of large, xylosylated *N*-glycans (Fig. S6; compare panels A and B), along with a relative increase in xylosylated *N*-glycans overall (Fig. S6, panel C). This suggests an indirect effect on *N*-linked glycan biosynthesis, potentially related to donor substrate availability (see Discussion).

**Fig 4:**
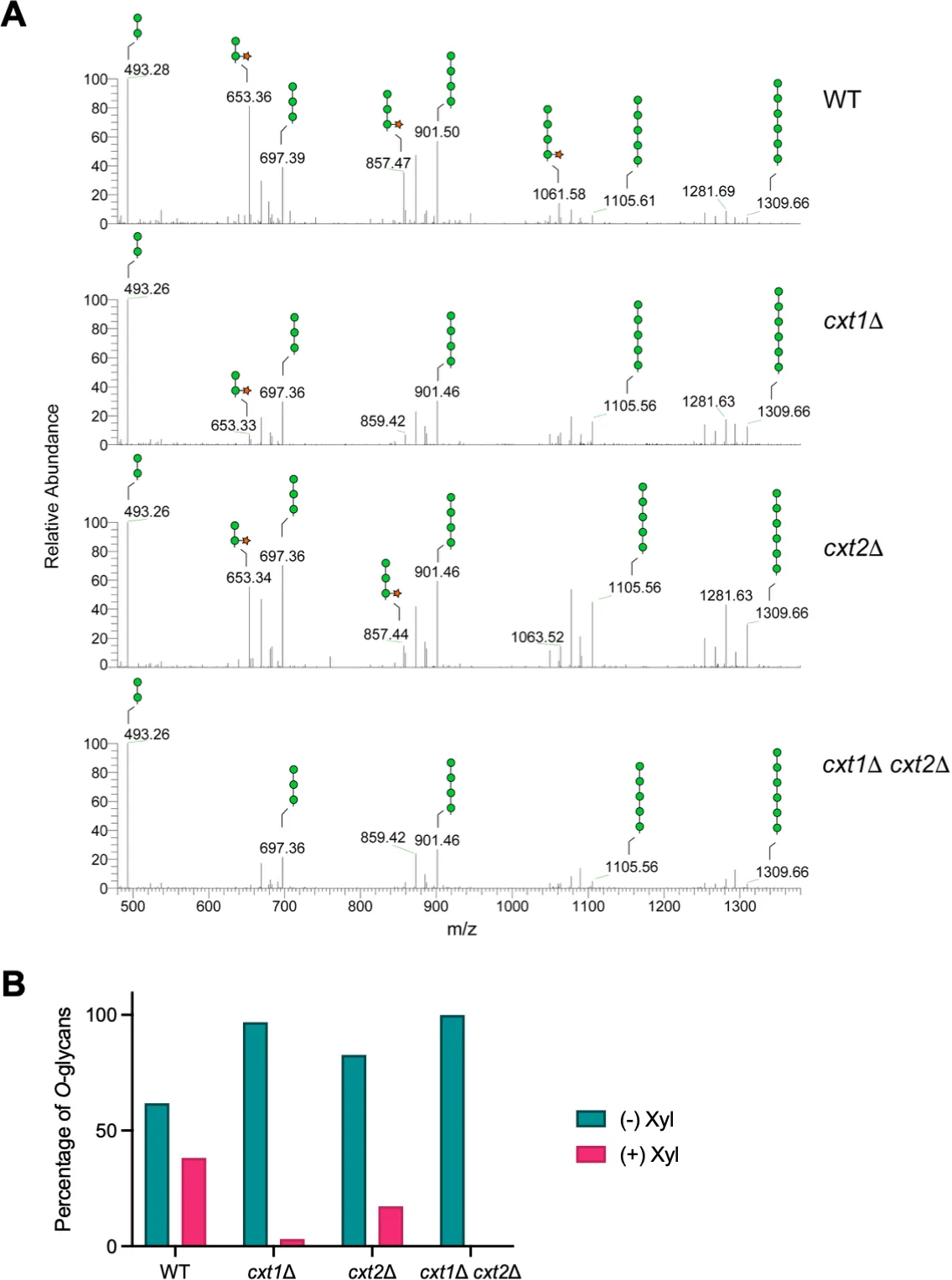
Cxt1 and Cxt2 mediate *O*-glycosylation. (**A**) Mass spectrometry spectra of *O*-linked glycans isolated from the indicated *C. neoformans* strains, showing the *m*/*z* region of the major *O*-linked glycans. Green circle, mannose; orange star, xylose. (**B**) Relative abundance of *O*-linked glycans ± xylose.

*O*-linked glycans of *C. neoformans* consist of a linear chain of α-linked mannoses, which may be modified with xylose on the reducing terminal mannose residue (Fig. S1E) (45, 47). To assess the roles of Cxt1 and Cxt2 in the synthesis of these structures, we analyzed *O*-glycans isolated from wild type, *cxt1*Δ, *cxt2*Δ, and *cxt1*Δ *cxt2*Δ. Notably, although the effect was greater in *cxt1*Δ, both single mutants showed a significant reduction in xylose-containing structures, including Xyl-Man_2_, Xyl-Man_3_, and Xyl-Man_4_ (Fig. 4A, peaks at 653.36, 857.47, and 1061.58 m/z, respectively; quantified in Fig. 4B). The double mutant showed complete loss of these structures (Fig. 4B). We conclude that, together, Cxt1 and Cxt2 mediate all xylose modifications of *O*-linked glycans in *C. neoformans*.

### Both β-1,2-xylosyltransferases are needed for capsule synthesis

**Fig 5:**
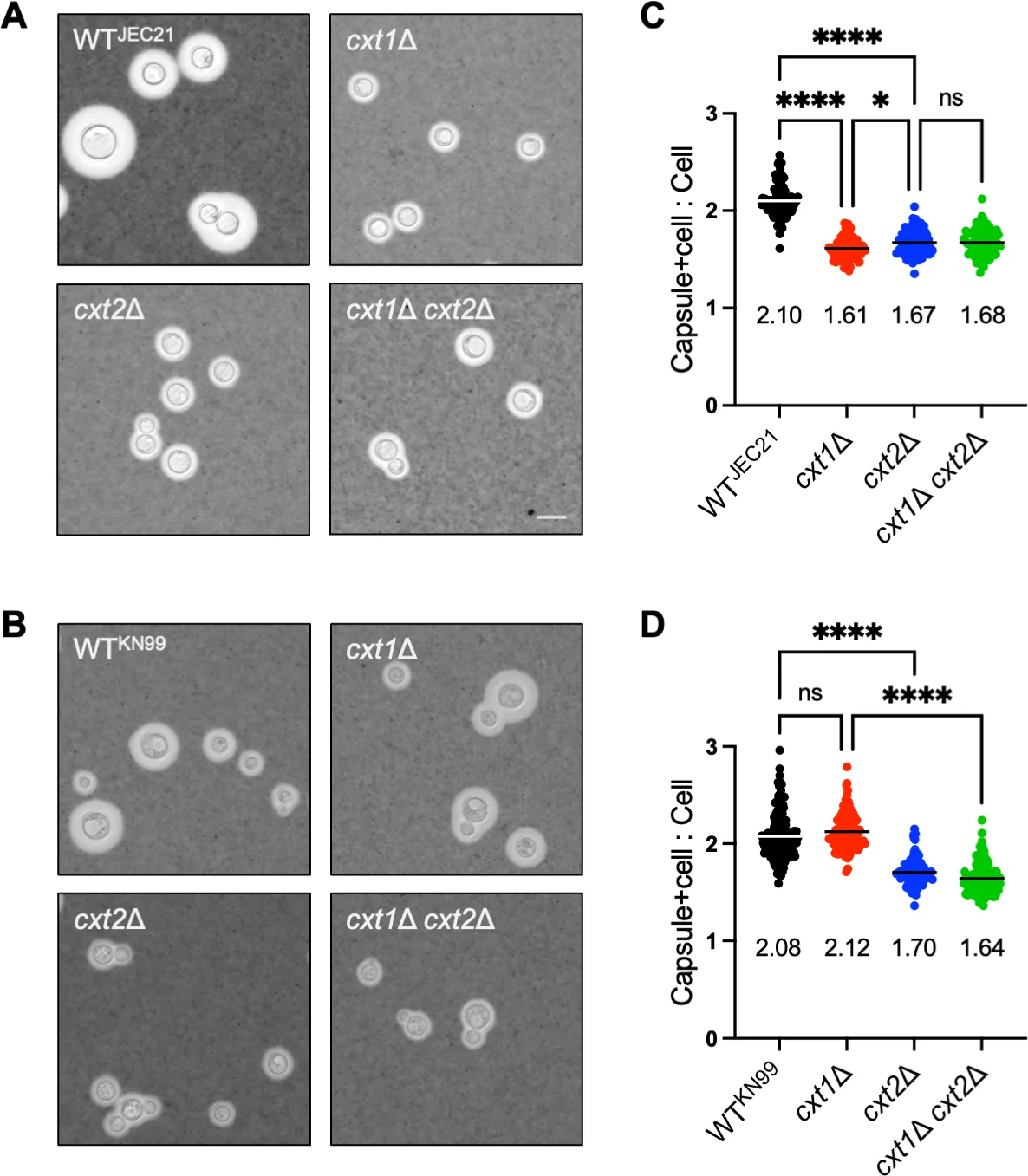
Cxt1 and Cxt2 influence capsule synthesis. (**A and B**) Representative images from India ink negative staining of JEC21 (**A**) and KN99 (**B**) strains grown in host-like conditions. All images are at the same scale; scale bar, 10 μm. (**C and D**) Values and means for total radius (capsule + cell) relative to cell radius alone, for at least 80 cells. *, *P* = 0.02; ****, *P* < 0.0001 by ordinary one-way ANOVA with Tukey’s multiple comparison test.

We previously found that Cxt1 acts in the synthesis of capsule polysaccharides: mutant strains exhibited an approximately 30% reduction of xylose in GXM and near-complete loss of mannose-linked xylose in GXMGal (38). However, we did not examine overall capsule size. To do this, we grew wild-type, *cxt1*Δ, *cxt2*Δ, and *cxt1*Δ *cxt2*Δ strains in “host-like” conditions that induce capsule expansion (here defined as DMEM tissue culture medium, 37°C, 5% CO_2_), where capsule synthetic enzymes might be most active (6, 48). We then visualized capsules by negative staining and measured their thickness. In JEC21, loss of either Cxt1, Cxt2, or both similarly reduced capsule size (Fig. 5A and C), suggesting that they play redundant roles in capsule synthesis in this background. Capsule imaging by transmission electron microscopy is consistent with this observation (Fig. S7A). Furthermore, the capsule fibers of the mutants appear twisted and short, similar to cells deficient in UDP-xylose synthesis or transport (32, 49). In KN99, both *cxt2*Δ and *cxt1*Δ *cxt2*Δ also had significantly smaller capsules (Fig. 5B and D). However, *cxt1*Δ capsules resembled those of the wild type, suggesting that Cxt2 is the predominant β-1,2-xylosyltransferase in capsule synthesis in this background. To further examine the physical properties of these capsule polysaccharides, we immunoblotted shed capsule material after agarose gel electrophoresis, using the anti-GXM antibody 18B7 (Fig. S7B). GXM from JEC strains migrated similarly in all samples although *cxt1*Δ and *cxt1*Δ *cxt2*Δ showed slightly stronger antibody binding. In KN99, both single mutants resembled WT, while *cxt1*Δ *cxt2*Δ exhibited altered migration and reduced antibody binding.

### β-1,2-Xylosyltransferases impact virulence

**Fig 6:**
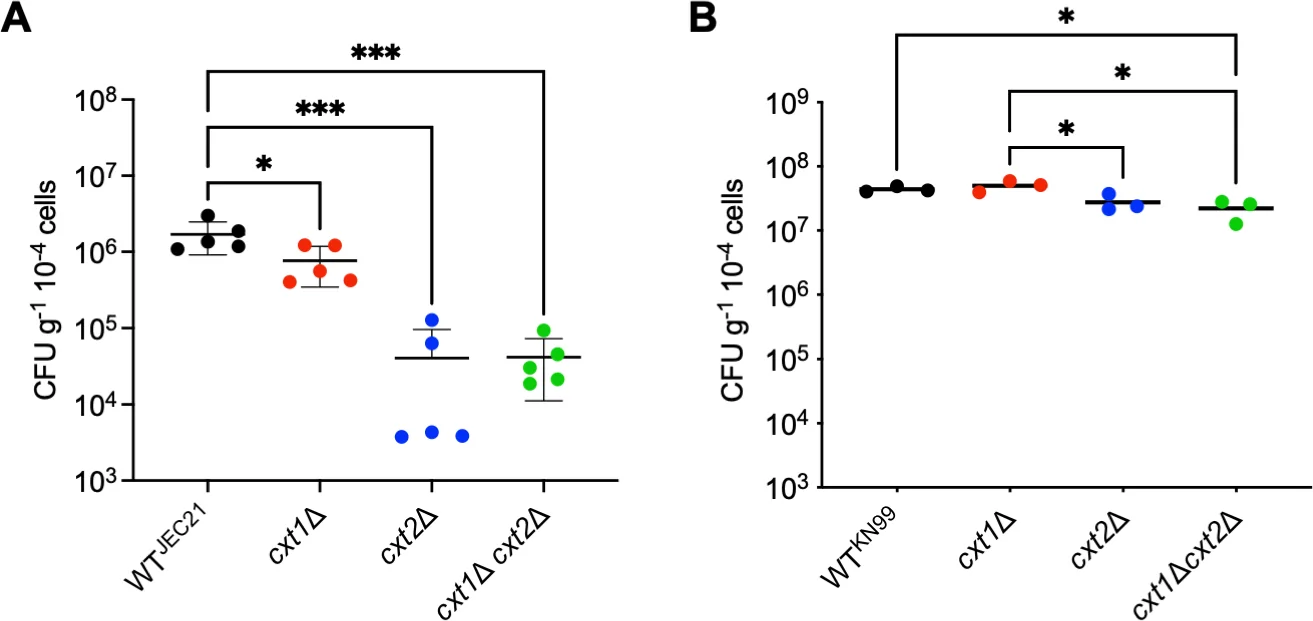
Impact of β−1,2-xylosyltransferases on virulence. Shown is lung burden of C57BL/6 mice sacrificed 12 days after intranasal infection with (**A**) 2.0 × 10^4^ cells in the JEC21 background or (**B**) 2.5 × 10^4^ cells in the KN99 background. Plots show the mean ± SD; each symbol represents one mouse. *, *P* < 0.05; ***, *P* ≤ 0.0001 by ordinary one-way ANOVA with Tukey’s multiple comparison test. Any comparisons not shown were not significant.

Strains with smaller capsules are often defective in animal models of cryptococcal infection (9). Indeed, our results using an inhalational model of murine infection mirrored the observed capsule phenotypes. Strains in the overall less virulent JEC21 background showed lower lung burden for all three mutants (Fig. 6A), each of which has reduced capsule, with greater reduction in the strains that also display stress sensitivity (*cxt2*∆ and the double mutant, Fig. 2 and Fig. S4). The corresponding KN99 strains showed more subtle reduction, although it also correlated with decreased capsule size (Fig. 6B). Together, these results suggest that Cxt2 plays a role in virulence (see Discussion).

### Cxt1 and Cxt2 colocalize in the Golgi apparatus

**Fig 7:**
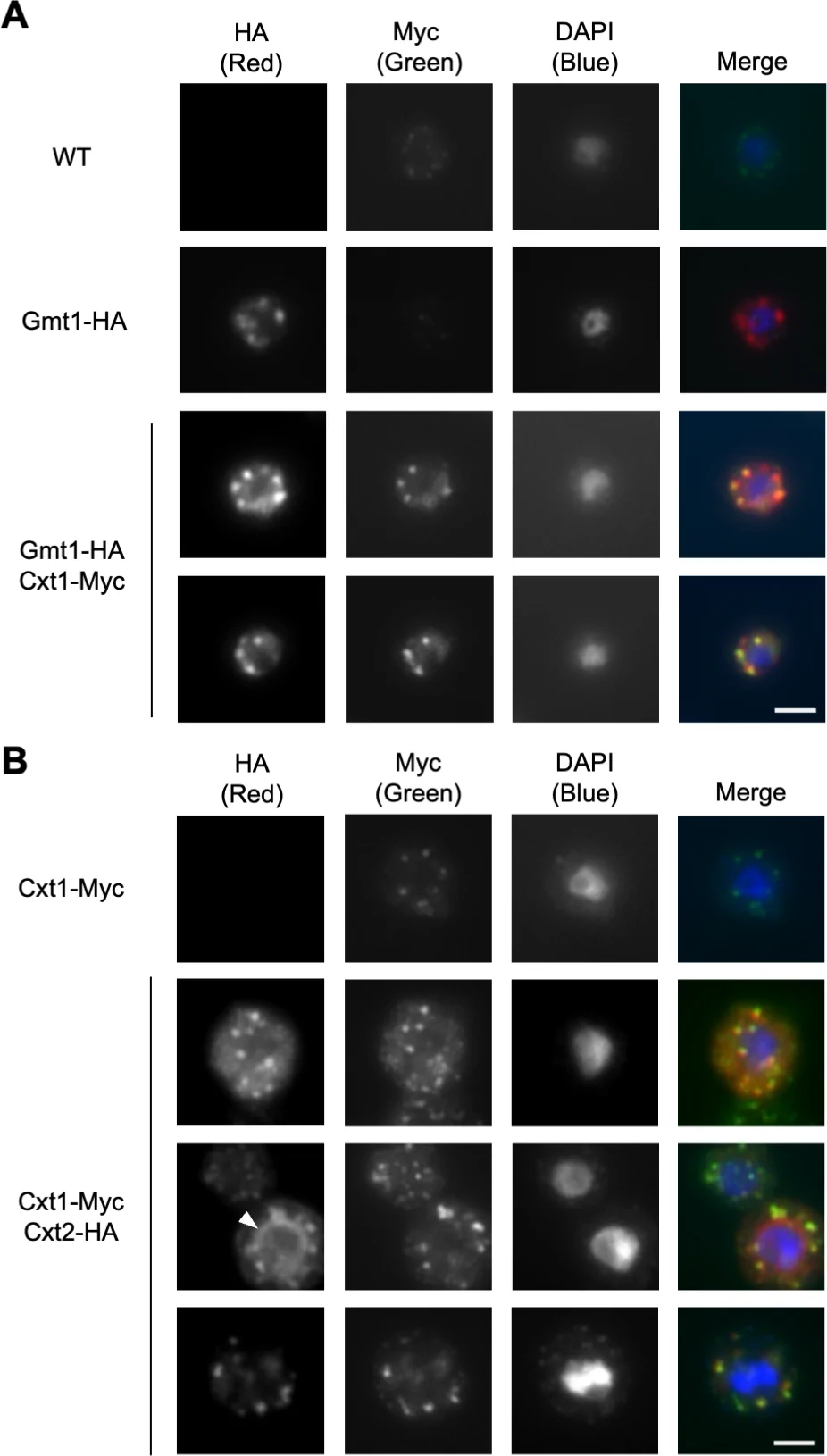
Localization of Gmt1, Cxt1, and Cxt2. Shown are representative immunofluorescence images of the indicated tagged strains in JEC21: (**A**) Gmt1-HA and Cxt1-Myc and (**B**) Cxt1-Myc and Cxt2-HA. Nuclei are stained with DAPI, and the white arrowhead indicates perinuclear signal. All images are at the same scale; scale bar, 2 μm. Each image was acquired at the same exposure and equally enhanced for visualization using ZenBlue software.

Our results show that Cxt1 and Cxt2 participate in multiple glycosylation processes that occur in the secretory pathway, including GIPC synthesis, protein glycosylation, and capsule polysaccharide synthesis (22, 50–53). Several of these are initiated in the endoplasmic reticulum and proceed in the Golgi apparatus (22); we previously found that UDP-xylose transporters, which provide the precursor for xylose addition, occur in both compartments (32). To investigate the subcellular localization of Cxt1 and Cxt2, we epitope-tagged each protein. By immunofluorescence microscopy, Cxt1-Myc, expressed at the native locus under its endogenous promoter, colocalized with the GDP-mannose transporter Gmt1 (32, 54), indicating Golgi localization (Fig. 7A). We also compared Cxt1 localization to that of Cxt2-HA, driven by the actin promoter. Both proteins localized in large puncta distributed throughout the cytosol; in most of these puncta, the tagged proteins colocalized (Fig. 7B). We also occasionally saw the Cxt2 signal as a perinuclear ring, suggestive of ER localization (Fig. 7B, white arrowhead), although this may reflect the overexpression required for detection.

### Cxt1 and Cxt2 do not interact

There is precedent in yeast for protein-protein interactions between glycosyltransferases, which may contribute to the efficiency of glycoconjugate synthesis (55, 56). Given that Cxt1 and Cxt2 colocalize, we wondered if they might physically interact. To test this, we performed coimmunoprecipitation studies with a strain expressing both Cxt1-HA and Cxt2-FLAG, each under its native promoter. However, we found no evidence of interaction between these proteins, whether cells were grown in yeast extract-peptone-dextrose (YPD) or host-like conditions (Fig. S8).

### Cxt1 and Cxt2 are differentially expressed

**Fig 8:**
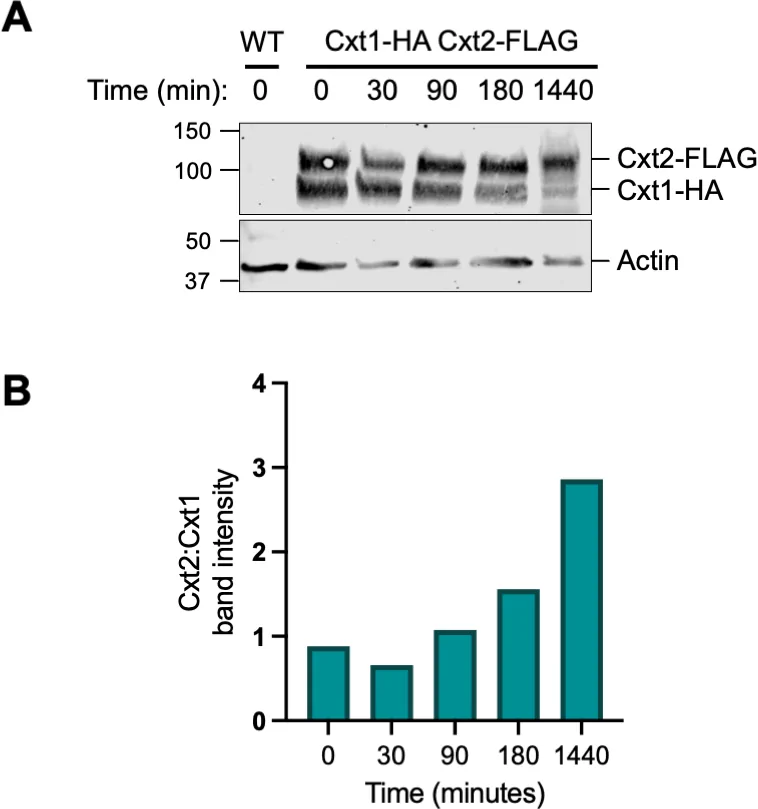
Cxt1 and Cxt2 protein expression. (**A**) Immunoblot of protein lysates from wild-type KN99 and Cxt1-HA Cxt2-FLAG strains using anti-HA (1:5,000), anti-FLAG (1:500), and anti-actin (1:1,000) antibodies. Protein (30 µg) was loaded per lane. Expected sizes: Cxt2-FLAG, 95 kDa; Cxt1-HA, 83 kDa; actin, 42 kDa. (**B**) Ratio of Cxt2:Cxt1 protein measured as band intensity normalized to actin. The immunoblot and quantitation shown are representative of two experiments, each with two independent strains.

Differential expression might also explain the distinct roles of Cxt1 and Cxt2. We previously published RNA-seq expression data for cells grown in host-like conditions over time (48). Analysis of this data set showed a modest increase over time in the expression of *CXT2*, with little change in that of *CXT1* (Fig. S9A). When we directly examined protein in the same conditions, we found that Cxt2-FLAG levels remained relatively steady, while Cxt1-HA decreased over time (Fig. 8A; Fig. S9B). This results in a relative increase in the ratio of the two signals over time (Fig. 8B) (see Discussion).

## DISCUSSION

The unique glycans of *Cryptococcus* play important roles in its biology and virulence. In previous studies, we identified Cxt1 as a β-1,2-xylosyltransferase that acts in the synthesis of GIPCs and capsule polysaccharides (37, 38, 44). Here, we have identified a second β-1,2-xylosyltransferase, Cxt2 (Fig. 1), and probed the biological roles of both enzymes.

Our most striking finding was that Cxt1 and Cxt2 together mediate all detectable xylose addition to *O*-glycans (Fig. 4), with Cxt1 playing the larger role. *O*-glycosylation is a highly conserved post-translational modification (57) with roles in protein folding, function, quality control, localization, and secretion (58–62). This process starts in the ER, where mannose is transferred from dolichol-phosphate-mannose to serine or threonine residues on polypeptides (63). In the Golgi, *O-*glycans are further extended by mannosyltransferases, forming linear structures of one to six α-mannose (up to six residues in our studies). In *Cryptococcus*, xylose is added to the first mannose residue at the reducing end of some of these *O*-mannosylated glycans (45). Cxt1 and Cxt2, which both localize in the Golgi (Fig. 7), are well positioned to participate in this process.

Inhibition of *O*-mannosylation activates the cell wall integrity pathway, unfolded protein response, and ER-associated protein degradation (59). In *Cryptococcus*, mutants defective in *O-*linked extension show reduced cell wall integrity signaling, stress resistance, virulence, and induction of the host immune response (45, 46). The phenotypic defects in *cxt1*Δ and *cxt2*Δ are more subtle, commensurate with the loss of only one residue.

Multiple studies report β-1,2-linked xylose in cryptococcal *N*-glycans (34, 44, 64), but neither Cxt1 nor Cxt2 seems to be directly involved in this process (Fig. 4) (44). We did find subtle differences at the population level (Fig. S6), but these changes may be indirect. For example, it may be that the absence of both Cxt1 and Cxt2 increases availability of UDP-xylose, the donor for xylose addition to *N*-glycans, explaining the higher fraction of *N*-glycans with this modification.

The polysaccharide capsule is a key feature in cryptococcal virulence. Cxt1 and Cxt2 are the first glycosyltransferases implicated in capsule synthesis to be localized, and are both found in the Golgi (Fig. 7). This supports our model of capsule synthesis occurring in that compartment (23). Cxt2 impacts capsule thickness in both strain backgrounds analyzed, while Cxt1 has less of a role in KN99 strains (Fig. 5). This corresponds with virulence in a mouse model of infection, where mutants lacking *CXT2* demonstrate reduced virulence relative to wild type (Fig. 6). Overall, our findings suggest that Cxt2 is more important for virulence than Cxt1.

Some mutant phenotypes, such as stress resistance (Fig. 2 and Fig. S4), differ by serotype. JEC21 strains unsurprisingly exhibit more severe defects, as *C. deneoformans* is generally less virulent and thermotolerant relative to *C. neoformans* (19, 20). Other reports have also noted variation in phenotypes dependent on strain background (65, 66). It is interesting that although the Cxt proteins have similar biochemical activity in both serotypes, the strain background ultimately dictates their functional impact on cell growth and virulence.

Some phenotypes we observed rely predominantly on one Cxt protein, as with the dependence of GIPC modification on Cxt1 (Fig. S5). For others, deletion of either gene yields a similar phenotype, as for capsule size in JEC21 (Fig. 5A and C). In still others, loss of both proteins is required to manifest the maximal phenotypic defect, as with *O*-glycosylation (Fig. 4). Together, these results suggest that the two proteins play distinct yet overlapping roles. This raised the questions of their relative expression and potential interaction, which we also pursued.

Analysis of gene expression over time, using our previously published data (48), suggested that while *CXT1* is the more highly expressed of the two genes (Fig. S9A), *CXT2* expression increases over time in host-like conditions, further supporting its role in virulence. RNA-seq of JEC21 grown at 30°C vs 37°C by Wallace et al. revealed similar patterns of expression (67). Interestingly, protein expression does not keep pace with gene expression for either one, with Cxt2 holding steady over time while Cxt1 decreases (Fig. 8A). This could be due to mRNA stability, processing, or efficiency of translation. (Protein amounts cannot be directly compared because different antibodies were used to detect them.) Despite their colocalization, we did not detect any direct interactions between Cxt1 and Cxt2, although they may interact indirectly or transiently (Fig. S8).

Overall, we found that Cxt1 and Cxt2 impact xylose addition to multiple major cryptococcal glycoconjugates, including GXM, GXMGal, GIPCs, and protein *O*-glycans, but are not directly involved in the synthesis of protein *N*-glycans. This suggests that *C. neoformans* encodes at least one other β-1,2-xylosyltransferase. Although we did not detect such an enzyme in our *in vitro* assays (Fig. 1), it may be lowly expressed in the rich growth conditions used for these assays or require a different acceptor than the α-1,3-mannobiose provided.

Xylose modification of proteins is known to impact the human immune response. In the model plant *Arabidopsis*, decreased exposure of xylose on *N-*glycans leads to reduced immunogenicity in allergy patients (68). In *Cryptococcus*, mutants with a higher proportion of xylose-containing *O-*glycans have enhanced immunogenicity relative to wild-type strains and vice versa (35). Conversely, loss of UDP-xylose synthesis (and thus xylose modification) leads to increased uptake by host macrophages (69) and enhanced complement activation (70). Strain-dependent variation in cryptococcal capsule composition, much of which involves altered xylose modification, also elicits different immune responses (71). Although uptake of *cxt1*Δ and *cxt2*Δ by host macrophages is unaltered relative to wild type (69), it will be interesting to investigate the impact of xylose modification on other aspects of the host immune response.

In this study, we examined the enzymatic activity and biological relevance of two β-1,2-xylosyltransferases, Cxt1 and the newly discovered Cxt2. These two enzymes play overlapping but distinct roles in cryptococcal biology, which interestingly differ between cryptococcal serotypes, highlighting the importance of utilizing multiple strain backgrounds. Our experiments resolve previously unknown components of cryptococcal glycan synthesis and identify the enzymes responsible for *O*-glycan xylose modification, moving us closer to a deep understanding of the synthesis of the unique glycoconjugates of *Cryptococcus*.

## MATERIALS AND METHODS

### Strain construction and growth

Strains used in this study are listed in Table S2, and their construction is detailed in the Supplemental material. All strains used in this study are mating type alpha. For both *C. neoformans* and *C. deneoformans* strain sets, deletion mutants and wild-type strains were otherwise isogenic products of mating. For all experiments, cryptococcal strains were grown on YPD plates for 2 days at 30°C. For liquid cultures, single colonies were inoculated into YPD and grown overnight at 30°C, with shaking. For capsule induction in host-like conditions, overnight YPD cultures were washed in PBS and grown in high-glucose DMEM (Sigma, D6429) at 10^6^ cells/mL for 24 h at 37°C and 5% CO_2_, without shaking.

### Crude membrane preparation and xylosyltransferase activity assays

Crude membrane preparations and xylosyltransferase activity assays were performed as we previously described (36). Overnight YPD cultures were resuspended in 100 mM Tris (pH 8.0) and 0.1 mM EDTA and disrupted by bead beating. Unbroken cells and debris were removed by centrifugation (1,200 × *g*; 20 min), and the resulting supernatant fractions were subjected to further centrifugation (60,000 × *g*; 45 min). The 60,000 × *g* pellets were thoroughly resuspended in the same buffer; the membranes were resedimented in the same conditions; and the supernatants were discarded. The washed membrane pellets were solubilized with 1% Triton X-100 on ice for 30 min with brief vortexing every 5 min, then centrifuged again (60,000 × *g*; 30 min), and the supernatant fractions were recovered as solubilized crude membranes.

To assay xylosyltransferase activity, solubilized crude membranes were incubated with 57 nmol UDP-[^14^C]xylose and 8.5 mM α-1,3-mannobiose for 4 h at 20°C. To remove excess UDP-[^14^C]xylose and terminate the reaction, the assay mixtures were applied to AG2X-50 resin and eluted with deionized water. The eluates were centrifuged (13,000 × *g*; 5 min), and the supernatants, containing soluble reaction products, were collected. For Jack Bean α-mannosidase digestion, the products were treated for 18 h at 37°C as in reference 36. The ^14^C-labeled products were detected by TLC.

For TLC, reaction products were dried under nitrogen at 50°C, resuspended in 15 μL of 40% *n*-propyl alcohol, and applied to 20 × 20 cm Silica Gel-60 TLC plates. Plates were developed in 5:4:2 *n*-propyl alcohol:acetone:water for 2 h, allowed to dry, and then developed again for 2 h. After drying, standards were visualized by spraying with 0.2% orcinol in 75:15:10 ethanol:sulfuric acid:water and heating at 70°C for 5–10 min. Sample lanes were sprayed with Enhance surface autoradiography spray and then visualized by autoradiography.

### Phenotyping

Overnight YPD-grown cultures were adjusted to 10^7^ cells/mL in PBS. Three microliter aliquots of 10-fold serial dilutions were spotted on YPD agar without or with 1.2 M NaCl, 0.01% SDS, 2.2 mM calcofluor white, or 10% DMSO (calcofluor white vehicle control). Plates were incubated at 30°C or 37°C for 72 h.

### Glycoprotein preparation, oligosaccharide release, and mass spectrometry

Cell lysates were prepared as we previously described (47), with all steps performed at 4°C with ice-cold buffer. Dense YPD cultures were sedimented and resuspended in 100 mM Tris (pH 8.0) and 0.1 mM EDTA and then disrupted by bead beating. Samples were centrifuged (2,000 × *g*; 25 min) to remove unbroken cells and debris, and the crude lysate (supernatant) was solubilized with 1% CHAPS (final concentration) by rotating for 2 h at 4°C. Insoluble material was removed by centrifugation (75,000 × *g*; 45 min), and the extracts were dialyzed against 2 L ice-cold 50 mM ammonium bicarbonate buffer using 6–8 kDa Fisher brand regenerated cellulose dialysis tubing for 48 h (with buffer changes every 12 h). Dialyzed samples were lyophilized, then washed with ice-cold 80% acetone to remove residual detergent and other contaminants.

*O-*linked oligosaccharides were released by reductive β-elimination and permethylated as we previously described (72). *N*-linked oligosaccharides were released from lyophilized cell extract material by PNGase F digestion and permethylated as we previously described (73, 74). Samples were then analyzed by mass spectrometry as detailed in the Supplemental material.

Mass spectra for these studies are available in GlycoPOST under accession number GPST000698 (75). Glycomics data and metadata were obtained and are presented in accordance with MIRAGE standards and the Athens Guidelines (76, 77). Symbol and text nomenclature for the representation of glycan structures is according to the Symbol Nomenclature for Glycans (78). The explicit identities of individual monosaccharide residues are assigned based on known fungal biosynthetic pathways. Data annotation and assignment of glycan accession identifiers were facilitated by the GRITS Toolbox (79), GlyTouCan (80), and GlyGen (81, 82).

### Capsule analysis

Cell cultures were induced as described above, then washed, resuspended in PBS, and mixed with India ink (Higgins) in PBS (2:1 vol/vol) to visualize capsule by negative staining. Randomly chosen fields of cells were imaged on a ZEISS Axio Imager M2 fluorescence microscope. The areas of each cell body with and without capsule were measured by ImageJ; their radii were calculated for at least 80 cells per sample and plotted as a ratio.

### Virulence studies

Groups of three or five 6- to 8-week-old female C57BL/6 mice from Jackson Laboratory were anesthetized by subcutaneous injection of 120 μL of 10 mg/mL ketamine and 2 mg/mL xylazine in sterile PBS and intranasally infected with either 2.0 × 10^4^ cells (JEC21 background) or 2.5 × 10^4^ cells (KN99 background) in 50 μL PBS. Mice were monitored with care and humanely sacrificed 12 days post-infection; lungs and brains were harvested, homogenized, and plated on YPD agar to assess fungal organ burden. Colony-forming units were counted, normalized to organ weight, and analyzed by ordinary one-way ANOVA with Tukey’s multiple comparison test.

### Immunofluorescence

Immunofluorescence was performed as in reference 47. Briefly, cells were cultured overnight in minimal medium [0.17% (wt/vol) YNB without amino acids and (NH_4_)_2_SO_4_, 0.5% (wt/vol) (NH_4_)_2_SO_4_, 2% (wt/vol) glucose] at 30°C, fixed with 3.7% formaldehyde, and subjected to partial cell wall digestion before 1% Triton X-100 permeabilization, staining, and imaging as detailed in the Supplemental material.

### Protein extraction and immunoblotting

YPD-grown cells were transferred to high-glucose DMEM at 37°C and 5% CO_2_ and grown for 0, 30, 90, 180, or 1,440 min before protein extraction. Cells were sedimented, washed in cold MilliQ water, and resuspended in ice-cold immunoprecipitation lysis buffer (100 mM Tris [pH 7.6], 150 mM NaCl, 1 mM EDTA, 1% NP-40, and protease inhibitor cocktail [Sigma P8215]). Cells were next disrupted by bead beating (eight rounds of 1 min bead beating followed by 1 min on ice), and unbroken cells were removed by centrifugation (500 × *g*, 20 min, 4°C). The protein concentration of the resulting supernatant (cleared cell lysates) was determined by BCA (Pierce BCA Protein Assay Kit, 23225).

Protein samples were mixed with 5× SDS sample buffer containing β-mercaptoethanol and boiled for 5 min. Samples were resolved on 4%–15% SDS-PAGE gels and transferred to nitrocellulose membranes, which were blocked with 10% milk in TBS for 1 h, washed with TBS Tween-20, and incubated with primary antibodies (1:5,000 anti-HA antibody [Abcam, ab9110], 1:500 anti-FLAG [Millipore Sigma, F3165], and 1:1,000 anti-actin antibody [ThermoFisher, MA1-744]) overnight at 4°C. Membranes were washed with TBS Tween-20, incubated with secondary antibody (1:10,000 680RD goat anti-mouse IgG [LI-COR IRDye, 926-68070] or 1:10,000 800CW goat anti-rabbit IgG [LI-COR IRDye, 926-32211]) for 1 h at room temperature, washed with TBS, and visualized on a LI-COR 92120 Odyssey Infrared imaging system.

### Statistical methods

Statistical analysis was performed using Prism (GraphPad) for ordinary one-way ANOVA with Tukey’s multiple comparison test.

### Additional methods

Detailed methods for RNA interference, protein sequence alignment and structure analysis, glycolipid preparation and analysis, transmission electron microscopy, GXM immunoblot, and coimmunoprecipitation are in the Supplemental material.

## SUPPLEMENTAL MATERIAL

Supplemental Material mbio.00806-26-s0001.pdf Supplemental figures and text.

Table S1 mbio.00806-26-s0002.xlsx N- and O-glycan structures and details corresponding to Fig. 3 and 4.

Table S2 mbio.00806-26-s0003.xlsx Strains used in this study.

ASM does not own the copyrights to Supplemental Material that may be linked to, or accessed through, an article. The authors have granted ASM a non-exclusive, world-wide license to publish the Supplemental Material files. Please contact the corresponding author directly for reuse.
